# Amyloid pathology arrangements in Alzheimer’s disease brains modulate in vivo seeding capability

**DOI:** 10.1186/s40478-021-01155-0

**Published:** 2021-03-30

**Authors:** Claudia Duran-Aniotz, Ines Moreno-Gonzalez, Nazaret Gamez, Nelson Perez-Urrutia, Laura Vegas-Gomez,, Claudio Soto, Rodrigo Morales

**Affiliations:** 1 Department of Neurology, The University of Texas Health Science Center at Houston, 6431 Fannin, St. Houston, TX 77030 USA; 2 Center for Social and Cognitive Neuroscience (CSCN), School of Psychology, Universidad Adolfo Ibanez, Santiago, Chile; 3 Latin American Brain Health Institute (BrainLat), Universidad Adolfo Ibanez, Santiago, Chile; 4 Universidad de los Andes, Facultad de Medicina, Av. San Carlos de Apoquindo, 2200 Las Condes, Santiago, Chile; 5 Department of Cell Biology, Faculty of Sciences, University of Malaga-IBIMA, 29010 Malaga, Spain; 6 Networking Research Center On Neurodegenerative Diseases (CIBERNED), Madrid, Spain; 7 Centro Integrativo de Biologia Y Quimica Aplicada (CIBQA), Universidad Bernardo O’Higgins, Santiago, Chile

**Keywords:** Alzheimer’s disease, Amyloid-beta, Prions, Strains, Pathology

## Abstract

Amyloid-β (Aβ) misfolding is one of the hallmark pathological features of Alzheimer’s disease (AD). AD can manifest with diverse symptomatology including variable rates of cognitive decline, duration of clinical disease, and other detrimental changes. Several reports suggest that conformational diversity in misfolded Aβ is a leading factor for clinical variability in AD, analogous to what it has been described for prion strains in prion diseases. Notably, prion strains generate diverse patterns of misfolded protein deposition in the brains of affected individuals. Here, we tested the in vivo prion-like transmission features of four AD brains displaying particular patterns of amyloidosis. AD brains induced different phenotypes in recipient mice, as evaluated by their specific seeding activity, as well as the total amount of Aβ deposited surrounding vascular structures and the reactivity of amyloid pathology to thioflavin S. Our results support the notion that AD-subtypes are encoded in disease-associated Aβ. Further research exploring whether AD include a spectrum of different clinical conditions or syndromes may pave the way to personalized diagnosis and treatments.

**Supplementary Information:**

The online version contains supplementary material available at 10.1186/s40478-021-01155-0.

## Introduction

Alzheimer´s disease (AD) is a heterogeneous and multifactorial brain disorder and the most common form of dementia in elderly population. AD is characterized by progressive neuronal degeneration, leading to severe cognitive impairments [1, 2]. The most prominent pathological hallmarks of AD are the extracellular deposition of amyloid-β (Aβ) peptides in the form of plaques and the intracellular accumulation of hyper-phosphorylated Tau (p-Tau) protein as neurofibrillary tangles [3]. Aβ, the proteolytic product of a transmembrane protein known as amyloid precursor protein (APP), is attributed with leading roles in AD as mutations in APP [4] or proteins linked to its proteolytic processing are associated with disease inheritance [5, 6]. In addition, strong evidence suggest that the misfolding and deposition of Aβ leads to the accumulation of neurotoxic hyper-phosphorylated tau [7]. Other important AD lesions, such as loss of neurons, synapse disruption, axonal trafficking impairment, oxidative stress [8], and activation of chronic neuroinflammation [9] are also associated with pathogenic Aβ.

AD is clinically and pathologically diverse. Variable clinical features include age at disease onset, clinical duration of the disease, rate of cognitive decline, manifestation of motor impairments, among others [10]. Pathologically, Aβ accumulation follows a sequential/progressive anatomical distribution that is initiated in the neocortex and subsequently expands to allocortical regions, striatum, brain stem and cerebellum [11]. Regardless of this well-conserved deposition pattern, extracellular Aβ deposits can manifest in diverse arrangements, including neuritic cored plaques, diffuse (lake-like and fleecy deposits), cotton wool plaques, etc., in addition to cerebral amyloid angiopathy (CAA) [12, 13]. Also, Aβ deposits can be differentially distributed at cellular (intra-, extra-) or tissue (parenchyma or vessels) levels [14, 15]. Unfortunately, the contribution of each type of deposits to neuronal death, brain inflammation and AD phenotypes is not completely understood. The specific distribution of these lesions across AD patients are thought to play a key role in brain atrophy and may modulate the clinical manifestations observed across individuals afflicted by AD [16, 17].

Currently, the factors or events directing Aβ to deposit in specific patterns are not known. Various lines of evidence suggest that Aβ misfolds and spreads in AD brains following mechanisms that resemble the replication of infectious prion proteins (PrP^Sc^) [18–21]. In that sense, several prion-like properties have been attributed to Aβ, including conformation and dose-dependent propagation [22–24], and brain invasion after administering preformed aggregates by different routes [25–27]. An intriguing feature of prions involves their conformational “strain” diversity. Compelling evidence suggest that prions strains differ in the conformational arrangements that the constituents prion proteins acquire [28, 29]. Strain-specific prion infection may lead to clinically diverse diseases as observed in experimental animals [30, 31] and humans [32–34]. Prion strains also induce variable distribution of PrP^Sc^ and patterns of spongiform degeneration in the brain of diseased individuals [33, 35–37]. Some reports suggest that such variability also exist for misfolded Aβ peptides [15, 38–42]. Actually, conformational variability on AD-associated Aβ has been linked to rapid disease progression [43]. Experimental data demonstrate that misfolded Aβ aggregates propagate different structural motifs in susceptible mouse models, partially resembling *bona fide* strain-specific prion transmission [39–41]. Regardless of this evidence, additional research is needed to establish whether strain variation at the level of Aβ modulates AD clinical and pathological variability.

In this study, we analyzed the in vivo prion-like propagation of disease-associated Aβ from AD brains displaying different amyloid pathology. Our results show that these AD brains induce diverse pathological features in treated mice, as measured by the intrinsic ability of each AD brain to promote amyloid pathology (seeding activity), and the particular vascular and thioflavin S positive pathology they induced. Our results suggest that different arrangements of misfolded Aβ template the propagation of unique pathological features associated with AD.

## Materials and methods

### Human tissues

Brain samples (frontal cortex) used in this study were collected *post mortem* from four clinically diagnosed AD patients and one non-demented individual. Left hemisphere was frozen and used to generate the inoculum. The right hemisphere was fixed in formaldehyde 37%. Both brain areas were dissected in their different anatomical components. Dissected fixed tissue was processed in increasing graded ethanol, embedded in paraffin and sliced. Specific information from each donor is provided in Table 1.

**Table 1:** Relevant information of brain donors

Code	Sex	Age (y/o)	Diagnosis	Cause of death
60129	Male	84	AD	Probable myocardial infarction
60068	Female	69	AD	Pulmonary
60649	Male	65	AD	Alzheimer disease
51486	Female	79	AD	Renal failure
58652	Male	59	Non-demented	Cardiopulmonary arrest

### Transgenic mice

APP/PS1 transgenic mice were used in this study. These mice over-express the human version of amyloid precursor protein (APP) harboring the Swedish double mutation (K670M and N671L) and the human presenilin-1 protein with the deltaE9 mutation (PSEN1-ΔE9) [44]. Treated animals were housed in groups of up to 5 in individually ventilated cages under standard conditions (22 °C, 12 h light–dark cycle) receiving food and water ad libitum. 4–6 animals per experimental group were used as indicated in each section. Males and females were indistinctly used (overall 42% males, 58% females). All animal experiments were carried out in accordance with the National Institutes of Health regulations and IACUC guidelines and approved by the committee of animal use for research at the University of Texas Health Science Center at Houston.

### Preparation and characterization of human brain inocula

Frozen frontal cortex samples were homogenized using an automatic homogenizer (Precellys 24-dual, Bertin Instruments) at 10% (w/v) in PBS containing a cocktail of protease inhibitors (Roche Diagnostics GmbH). Resulting homogenates were stored at − 80 °C until used for animal injection or ELISA measurements of Aβ peptides (as explained below).

### Intra-cerebral inoculation of brain extracts

 ~ 30 days-old APP/PS1 mice were intra-cerebrally injected with 10 μL of 10% (w/v) brain homogenates from AD patients or a non-demented individual. For injection, animals were anesthetized using isoflurane and fixed to a mouse stereotaxic frame. Unilateral injections were performed at a single point in the right hippocampal area using the following coordinates as measured from Bregma: antero- posterior (AP), −1.8 mm; medio-lateral (ML), −1.8 mm; dorso-ventral (DV), −1.8 mm. Immediately after treatment, skin was closed using surgical suture. The injection was conducted at a rate of 0.5 µL/min and the needle was left in place for 3 min before retraction. Animals were placed on a thermal pad until recovery and monitored daily for several days. Mice were sacrificed by CO_2_ inhalation at ~ 150 days after inoculation. Brains were removed and stored in 10% formalin for histological studies.

### Histological analysis

10-μm-thick serial slices from all animal groups or human subjects were processed in parallel for histological analyses. Mouse-derived specimens were processed from lambda 0 to lambda −4 mm. For immunohistochemistry, sections were deparaffinazed and hydrated and the endogenous peroxidase activity was blocked with 3% H_2_O_2_/10% methanol in PBS for 20 min. After formic acid epitope retrieval (formic acid 85% for 5 min), primary antibody 4G8 was incubated over night at a 1:1,000 dilution at room temperature (Covance, Princeton, NJ). HRP-linked secondary sheep anti-mouse antibody at a 1:1,000 dilution (GE Healthcare, Little Chalfont, UK) was incubated for 2 h at room temperature. Peroxidase reaction was visualized using DAB Kit (Vector, Burlingame, CA) following the manufacturer’s instructions. Finally, sections were dehydrated in graded ethanol, cleared in xylene, and cover-slipped with DPX mounting medium (Innogenex, San Ramon, CA). Additional brain sections were incubated with Thioflavin-S (ThS) solution (0.025% in 50% ethanol) for 8 min, and coverslipped with DPX. All samples were analyzed using an inverted microscope for bright field and an epifluorescent microscope for ThS staining (DMI6000B, Leica, Buffalo Grove, IL, USA) and then quantification analysis was performed using the ImagePro software (Rockville, MD, USA). Five tissue slices per animal/staining, taken every 10 slices were used for image analysis quantifications. For specific quantification of vascular Aβ deposition, slices stained with 4G8 were quantified for brain-parenchymal blood vessel associated signals as described before [45] in the cerebral cortex. Leptomeningeal deposits were not included in these analyses. Aβ and ThS burdens were defined as the area of the brain labeled per the total area analyzed. Burden quantification was performed by an investigator blinded to the experimental groups. Vascular deposition in the hippocampus of treated mice was scarce and was semi-quantitatively evaluated in a present/absent manner (Additional file 1: Table 1).

For double staining, human sections were auto-fluorescence blocked with Autofluorescence Eliminator Reagent (Millipore 2160), subjected to antigen retrieval using 10 mM citrate buffer pH 6.0 for 30 min at 80˚C and blocked with 5% BSA to avoid non-specific binding. Thereafter, samples were incubated with rabbit anti-smooth muscle actin (SMA) (Proteintech, Illinois, USA) primary antibody overnight at a 1:1,000 dilution at room temperature. Consecutively, sections were incubated with mouse anti-4G8 antibody (as stated previously) or ThS solution (0.025% in PBS for 8 min). Primary antibodies were visualized with anti-mouse Alexa Fluor488 and anti-rabbit Alexa Fluor 594 at a 1:1,000 dilution for 1.5 h (ThermoFisher, USA). Finally, samples were washed and cover-slipped with FluorSave mounting medium (Millipore Sigma, Massachusetts, USA). Analyses of vascular amyloidosis in mice’s brains were done as explained above but using 4G8 antibody instead of ThS.

### ELISA quantification of Aβ species

Brain extracts from AD patients and a non-demented individual were subjected to a previously described serial Aβ extraction protocol [22, 46, 47]. Briefly, 10% (w/v) brain homogenates were centrifuged in L100K ultracentrifuge tubes (Beckman-Coulter, Brea, CA) at 32,600 rpm for 1 h at 4 °C, using a 42.2 Ti rotor. Supernatants were saved (PBS fractions) and pellets were resuspended in 2% sodium dodecyl sulfate (SDS) by pipetting and sonication (using a bath sonicator) until complete disruption. Samples were subjected to the same centrifugation procedure explained above. The resulting supernatants were collected (and saved as SDS fractions), pellets resuspended in 70% formic acid (FA, Fisher Scientific, Waltham, MA) and sonicated in a bath sonicator until complete disruption. Then, samples were centrifuged for 30 min using the same conditions explained above and supernatants were collected (FA fractions). FA fractions were 20-fold diluted on 1 M Tris buffer, pH 11 (Sigma-Aldrich, St. Louis, MO) to neutralize pH. Aβ_42_ peptides present in these samples were measured by ELISA using a commercially available kit (KHB3442, Invitrogen, Carlsbad, CA). ELISA was performed following manufacturer’s instructions.

### Calculation of seeding activity ratio

Seeding activity ratio for each inoculum was calculated by dividing burden values present in each animal (cortex and hippocampus) by the Aβ burden present in each AD brain used for inoculation.

### Comparisons between different pathological features in treated mice

Each parameter measured in mice injected with AD brain extracts (Aβ burden and ThS burden in different regions, vascular amyloidosis and induction ratios) were normalized to a value of 1 considering the maximum averaged value among all groups for each parameter. Data obtained in this manner for all pathological assessments was combined in a single graph using Microsoft Excel.

### Statistical analyses

Data are expressed as means ± standard error of the mean (SEM). After confirming normal distribution with Skewness/Kurtosis statistic test, one way ANOVA was used to analyze differences in histological assays. Statistical analyses were performed using Graph Pad Prism 5.0 software. Statistical differences were considered significant for values of *p* < 0.05.

## Results

### Characterization of Aβ pathology in the brains of four AD patients

**Fig. 1:**
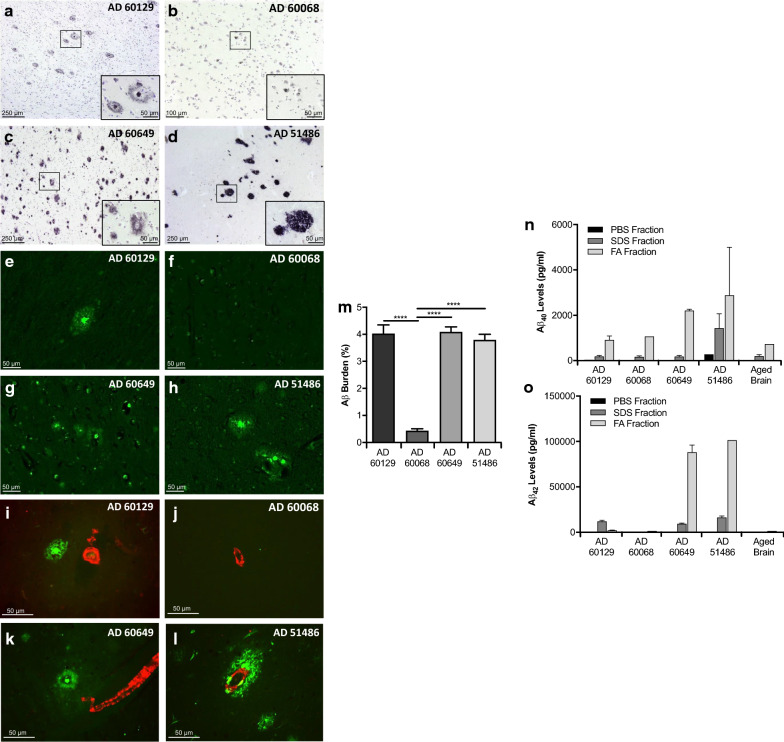
Characterization of four AD brains displaying different amyloid pathology. Brains from four clinically confirmed AD patients were characterized for Aβ deposition using immunohistochemistry and ELISA. **a**–**d** Brain slices from patients AD60129, AD60068, AD60649 and AD51486 were immunostained to assess Aβ deposition. Insets represent higher magnifications of representative amyloid plaques in each specimen. **e**–**h** Thioflavin S (ThS) staining for the same samples described above. Characterization for brain 58652 (non-demented) is provided in [46]. **g**–**j** Double staining using ThS (green) and anti-SMA antibody (red) was performed to study the presence of vascular Aβ in human samples. (M) Image analysis quantification (% burden) of Aβ pathology in samples AD60129, AD60068, AD60649 and AD51486. (N–O) Aβ_40_ and Aβ_42_ was serially extracted in PBS, SDS and FA as explained in Materials and Methods. Aβ peptides in each fraction were measured by human-specific Aβ ELISA kits. Data values were expressed as mean ± SEM. Data from (M) was obtained from multiple tissue slices and analyzed by one-way ANOVA followed by Tukey's multiple comparison post-test. *p < 0.05, **p < 0.01, ***p < 0.001, ****p < 0.0001

Aβ pathology in the brains of AD patients was assessed by histopathological and biochemical analyses. All four patients included in this study (AD60129, AD60068, AD60649 and AD51486) were clinically diagnosed for AD. Immunohistochemical staining against Aβ revealed strikingly different patterns of amyloid deposition among them (Fig. 1). Brain AD60129 displayed abundant neuritic and cored plaques and some diffuse aggregates (Fig. 1a). As expected, many of the deposits present in this sample were reactive against Thioflavin S (ThS, Fig. 1e), a dye binding compact amyloid structures [48]. AD60068 showed small intracellular aggregates but no parenchymal deposits were observed (Fig. 1b). This feature was unique among all specimens used in this study. No ThS-positive deposits were found in this brain tissue (Fig. 1f). AD60649 sample displayed abundant Aβ deposition albeit the majority of aggregates observed in this patient were less compact as the ones observed in sample AD60129 while also displaying abundant diffuse Aβ plaques (Fig. 1c). Here, ThS positive signals were observed in smaller but more abundant deposits (Fig. 1g). Finally, brain AD51486 displayed diffuse (cotton wool, Fig. 1d) and cored Aβ deposits with abundant cerebral amyloid angiopathy (CAA). The presence of CAA in this and all other AD samples was further characterized by immunohistochemical (IHC) analyses. Specifically, ThS positive Aβ deposits were assessed for co-localization with the smooth muscle actin (SMA) protein, a marker of blood vessels. Panels I-L in Fig. 1, and Additional file 1: Fig. 1, further confirm the strong CAA phenotype in sample AD51486 compared to all other specimens used in this study. Additional file 1: Fig. 1 additionally shows that occasional CAA was found only in sample AD60649 (a single event in all tissue slices analyzed). Vascular amyloidosis was absent in AD60129 and AD60068 brains. ThS positive amyloidosis in AD51486 was observed for both, parenchymal and vascular aggregates (Fig. 1h).

Aβ burden quantification of 4G8-inmunoreactivity by image analysis (Fig. 1m) showed similar values for samples AD60129, AD60068 and AD51486. Amyloid deposition in sample AD60068, displaying abundant intracellular Aβ accumulation, was considerable lower (~ ninefold) compared to all other samples. These results were confirmed by ELISA measurements of Aβ_40_ (Fig. 1n) and Aβ_42_ (Fig. 1o) after serial extraction in PBS, SDS and FA. Aβ_40_ levels measured in the FA-fractions were substantially higher in sample AD51486 compared to all other specimens, in line with the higher vascular amyloidosis observed for this sample in IHC analyses (Fig. 1l). Brain AD60649 also displayed high Aβ_40_ concentrations in the SDS- and FA- fractions. However, values collected in the SDS-fraction were not different compared to the ones in AD60129, AD60068 and the brain of a non-demented individual (sample 58652). As expected, levels of PBS-insoluble Aβ_42_ proteins displayed higher differences between AD specimens and the control brain. In agreement with the SDS- and FA- extracted Aβ_40_ levels, samples AD51486 and AD60649 contained the highest accumulation of this disease-associated peptide (~ 3–7 times) compared to sample 58652 (aged non-demented control). Sample AD60129 displayed lower PBS-insoluble Aβ_42_ concentrations compared to AD51486 and AD60649, although substantially higher compared with the control brain. In contrast, sample AD60068 did not show Aβ_42_ concentrations that differed from the non-demented control brain. Overall, the PBS-soluble Aβ_40_ and Aβ_42_ levels were not substantially different across all samples analyzed. A summary of the findings described in Fig. 1 is presented in Table 2.

**Table 2:** Summary of pathological features observed across AD patients

Code	Aβ deposits	Aβ burden	Vascular Aβ	Insoluble Aβ_40_	Insoluble Aβ_42_
60129	Cored plaques	High	Low	Low	Medium
60068	Intracellular	Low	None	Low	Low
60649	Diffuse and cored plaques	High	Low	Medium	High
51486	Diffuse (cotton wool)	High	High	High	High
58652	ND	ND	ND	Low	Low

*ND* notdetermined

The characterization presented above is a good example of the pathological variability displayed across the brains of AD patients. In that sense, extracts prepared from these AD tissues were used to test their intrinsic seeding activities in susceptible mouse models of cerebral amyloidosis.

### In vivo seeding activity of AD brains displaying different amyloid pathology

**Fig. 2:**
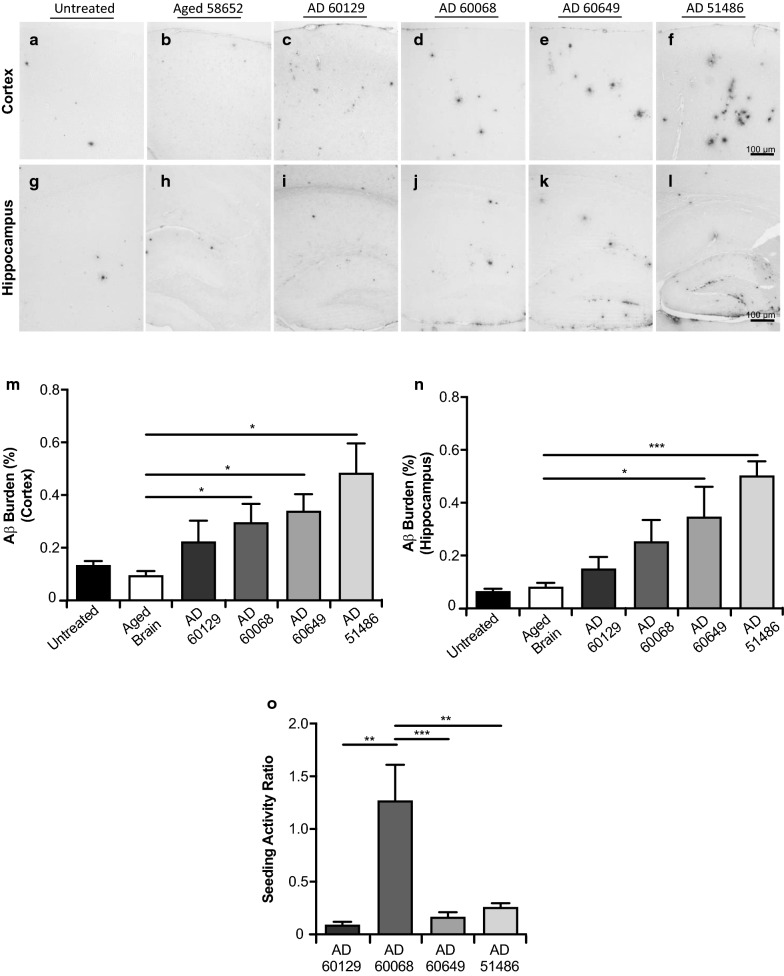
Aβ seeding activity of pathologically diverse AD brains. Brain cortex (**a**–**f**) and hippocampus (**g**–**l**) of mice inoculated with different brain extracts were analyzed for their content of Aβ deposits. Equivalent specimens from untreated mice were included as controls. **m**, **n** Image analysis quantification (% burden) of Aβ pathology in cortex (**m**) and hippocampus (**n**). **o** The intrinsic seeding capacity of each inoculum was calculated as the ratio between Aβ burden in each mouse and the burden in the inoculum used for treatment. N = 4–6/group. Data value were expressed as mean ± SEM. Data from M and N were analyzed by Student's t-test comparing the experimental groups with Aged Brain. Data from **o** were analyzed by one-way ANOVA followed by Tukey's multiple comparison post-test. *p < 0.05, **p < 0.01, ***p < 0.001

Extracts from AD brains displayed in Fig. 1 were intra-cerebrally inoculated in ~ 30 days old APP/PS1 mice. Animals were sacrificed 150 days after the treatment and brains collected for histopathological analyses (Fig. 2). Untreated animals (Fig. 2a, g) and mice injected with brain extract from an aged non-demented individual (58,652) (Fig. 2b, h) acted as negative controls. Brain slices from mice in the control groups displayed modest amyloid pathology, similarly as previously described [46, 49]. Image-analysis quantification of Aβ burden in cortex and hippocampus of mice in these two negative control groups did not display significant differences, suggesting that the non-demented brain does not carry Aβ seeding activity (Fig. 2m, n). On the contrary, most AD brains (AD51486, AD60649 and AD60068) induced significantly higher amyloid pathology compared to the control groups. Brain AD60129 displayed a positive trend in terms of amyloid deposition compared to controls, although this increase did not reach statistical significance. Amyloid deposition of mice treated with AD brains was arranged in the following pattern: AD51486 > AD60649 > AD60068 > AD60129.

In order to explore the intrinsic seeding activity of each AD brain, burden values present in each animal (cortex and hippocampus) were divided by the Aβ burden present in each corresponding AD brain used for inoculation (Fig. 2o). Surprisingly, the highest intrinsic seeding activity was found for brain AD60068, the one displaying the lowest Aβ accumulation burden. Using the same analysis, all other brains showed lower but similar seeding activities among themselves. These results show that amyloid seeding is independent of the total Aβ content, but a consequence of their specific pathological arrangements.

### Inoculum-dependent induction of vascular amyloid deposition

**Fig. 3:**
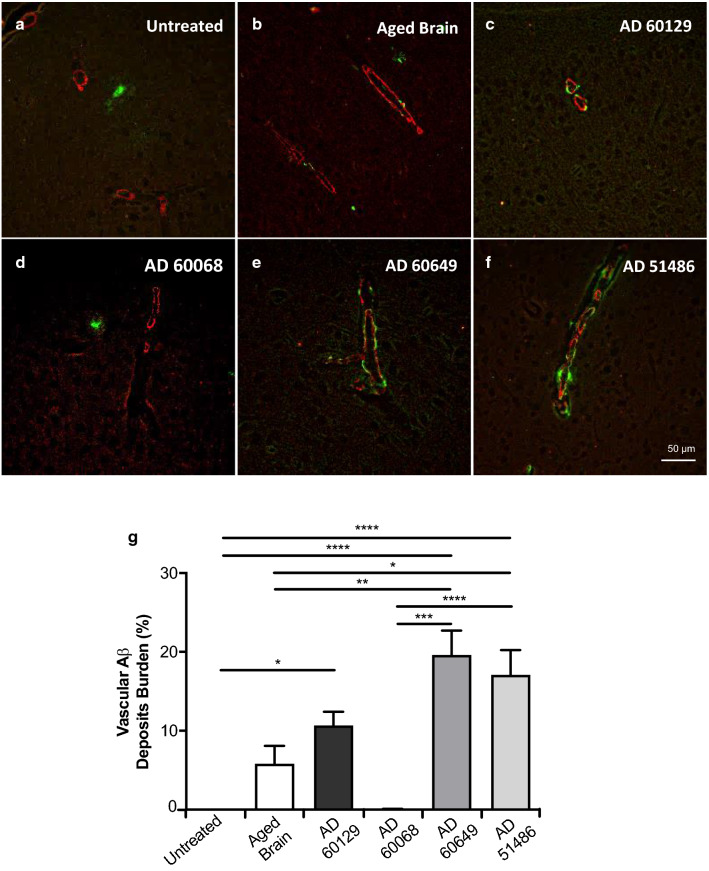
Vascular deposition in the brain of APP/PS1 mice challenged with AD brain extracts. **a**–**f** Double immunofluorescence staining for Aβ (green) and SMA (red) in mice’s brains inoculated with human AD brain extracts. **g** The extent of amyloid deposition associated with vessels was calculated as previously reported [45] using bright field images. Values for each group are expressed as the area of Aβ 4G8-inmunoreactivity in vascular structures divided by the total Aβ burden and multiplied by 100. N = 4–6/group. Data value were expressed as mean ± SEM. Data were analyzed by one-way ANOVA followed by Tukey's multiple comparison post-test. *p < 0.05, **p < 0.01, ***p < 0.001, ****p < 0.0001

Misfolded Aβ in AD can deposit in either the brain parenchyma or vasculature [14, 15]. CAA leads to deleterious events including microhemorrhages that may worsen clinical deterioration [50, 51]. Previous results show that exogenous Aβ seeding induce CAA in susceptible mice [25, 52]. Considering the variable vascular amyloidosis in the four AD samples included in this study, we assessed whether they differentially induced CAA in treated mice. When present, most vascular aggregation was found in the cerebral cortex (Fig. 3a–f). As expected, no vascular aggregates were observed in blood vessels of the brain parenchyma in untreated APP/PS1 mice, as these animals develop few CAA events only at advanced ages when brain amyloidosis is extensive (unpublished data). Surprisingly, the brain from the aged non-demented control individual induced some CAA in recipient mice (Fig. 3b). This result was unexpected, as the total Aβ burden in the brain of these mice was not significantly different compared to untreated controls. However, it is important to highlight that this inoculum had detectable levels of insoluble Aβ (Fig. 1n) that could alter some aspects of Aβ misfolding propagation in recipient mice. All AD samples, with the sole exception of AD60068 (displaying small distribution of mostly intracellular aggregates) induced vascular amyloidosis in blood vessels of the brain parenchyma of recipient mice (Fig. 3). Vascular amyloidosis in the AD60649- and AD51486-derived groups was significantly higher compared to the one found in mice receiving the control injectate and ranged from 23.7 to 19.4% of the total deposition in the brain cortex (Fig. 3g). These results are relevant, considering that the AD60068 homogenate displayed the highest intrinsic seeding capacity, and suggest that Aβ extracellular deposits may modulate the propagation of amyloidosis to vascular structures.

We also evaluated Aβ deposition in hippocampal blood vessels of all treated mice. CAA in this specific brain region was scarce and it was found in just a fraction of experimental mice (Additional file 1: Table 1). The presence of vascular amyloidosis in this brain region largely correlated with cortical values as it was found only in some animals treated with the AD60649 and AD51486 inocula. No vascular Aβ deposits were found for animals injected with the AD60129 brain extract, despite this injectate promoted vascular pathology in the cortex. This data further suggest that induction of brain amyloidosis is not linear and depends of the intrinsic properties of each inoculum.

### Prion-like induction of amyloid pathology induces diverse populations of Aβ deposits

**Fig. 4:**
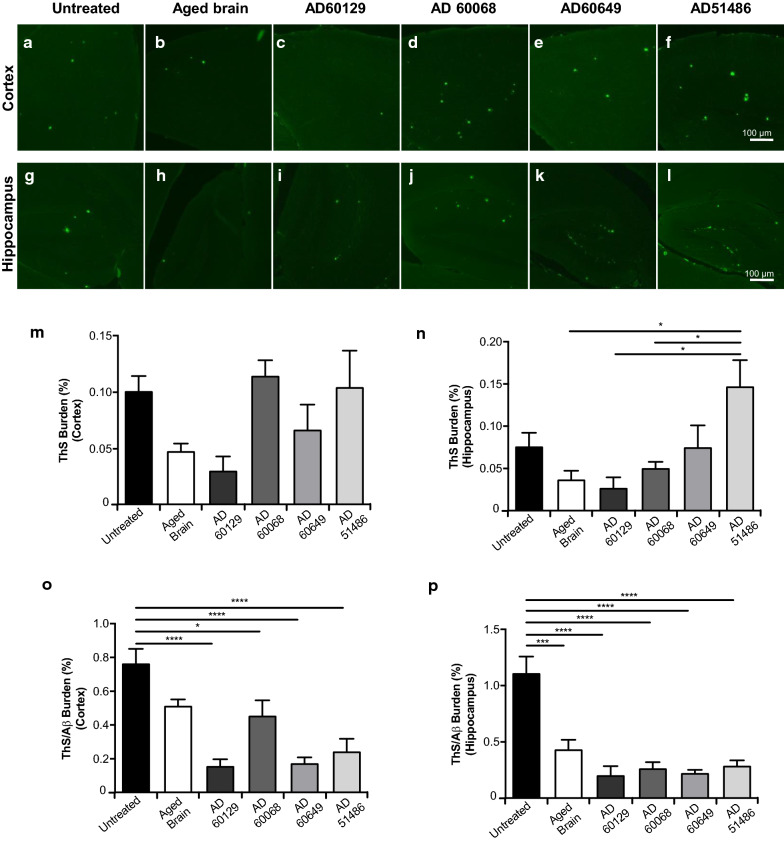
Thioflavin S staining in APP/PS1 mice intra-cerebrally inoculated with different AD brain specimens. Brain cortex (**a**–**f**) and hippocampus (**g**–**l**) from APP/PS1 mice injected with different human brain extracts were stained with ThS. Brains from untreated/age-matched mice (**a**, **g**) were included for comparisons. **m**, **n** Image analysis quantification (% burden) of ThS positive signals in cortex (**m**) and hippocampus (**n**) of APP/PS1 mice. **o**, **p** ThS values in each animal in cortex (**o**) and hippocampus (**p**) were divided by their corresponding Aβ values (calculated in Fig. 2) to estimate the ratio of ThS positive plaques. N = 4–6/group. Data value were expressed as mean ± SEM. Data were analyzed by one-way ANOVA followed by Tukey's multiple comparison post-test. *p < 0.05, ***p < 0.001, ****p < 0.0001

Compact amyloid aggregates can react to several dyes, such as thioflavin and Congo red [48]. For that reason, these reagents have been extensively used as pathological markers in both diagnostic and research contexts. Importantly, the degree of compactness displayed by amyloid aggregates can be used as a surrogate of pathological variability. We stained brain tissue of experimental and control mice with ThS and compared their pathological phenotypes at this level. Our results show that all groups displayed ThS positive Aβ deposits in both cortex and hippocampus (Fig. 4a–l). Cortical measurements showed a heterogeneous ThS-positive burden, albeit no significant differences were found across the groups (Fig. 4m). In the hippocampus, sample AD51486 induced significantly higher levels of ThS-positive deposits, in line with the highest overall amyloid induction generated by this particular sample (Fig. 4n).

Considering the different levels of total amyloidosis observed across the groups, we calculated the ratio between ThS values and 4G8-positive IHC to assess the distribution of plaques reactive to ThS compared to the total Aβ deposition present in each brain (Fig. 4o, p). We observed that most of the deposits found in untreated control mice were reactive against ThS. On the contrary, the ThS/4G8-reactivity ratios in animals inoculated with the AD and control brains were significantly lower in the hippocampus (Fig. 4o). In the cortex, all AD-inoculated brains exhibited different values compared to the untreated control, while the group induced with the control brain (58,652) was not significantly different, in line with vascular data displayed in Fig. 3. This data further suggests that brains from AD patients displaying differential pathology propagate inoculum-specific deposits in susceptible mice.

## Discussion

AD is linked to several pathological features, including Aβ and tau deposition, glial activation and brain atrophy [2]. Variations in some of these properties have been associated with clinical manifestations [15]. Considering Aβ misfolding and accumulation as an early event in AD, disparities at this level may be amplified downstream at pathological cascades resulting in various clinical symptoms [15–17].

Conformational strain diversity has been extensively described for infectious prions [29, 53, 54]. Rodent-adapted prion strains are known to generate distinguishable changes in terms of prion protein deposition and spongiform degeneration in the brain [29, 31, 36, 37, 54]. Prion strains can be discriminated at the molecular level by assessing several parameters such as their electrophoretic mobility after protease digestion, glycosylation profiles, and resistance to proteolytic digestion or denaturation [29, 55–57]. Similar characterizations on misfolded Aβ aggregates suggest that this disease-associated protein also displays conformational strain variations [39, 43]. The existence of “Aβ strains” has been attributed as the cause of rapid and slow cognitive decline observed across AD patients [43]. Structural changes have also been found in misfolded Aβ structures from patients afflicted by different AD types [58]. Aβ strains are able to propagate various conformations in in vitro and in vivo systems [39, 42, 58]. However, the biological significance of misfolded strain variation in AD’s Aβ is not well understood.

**Fig. 5:**
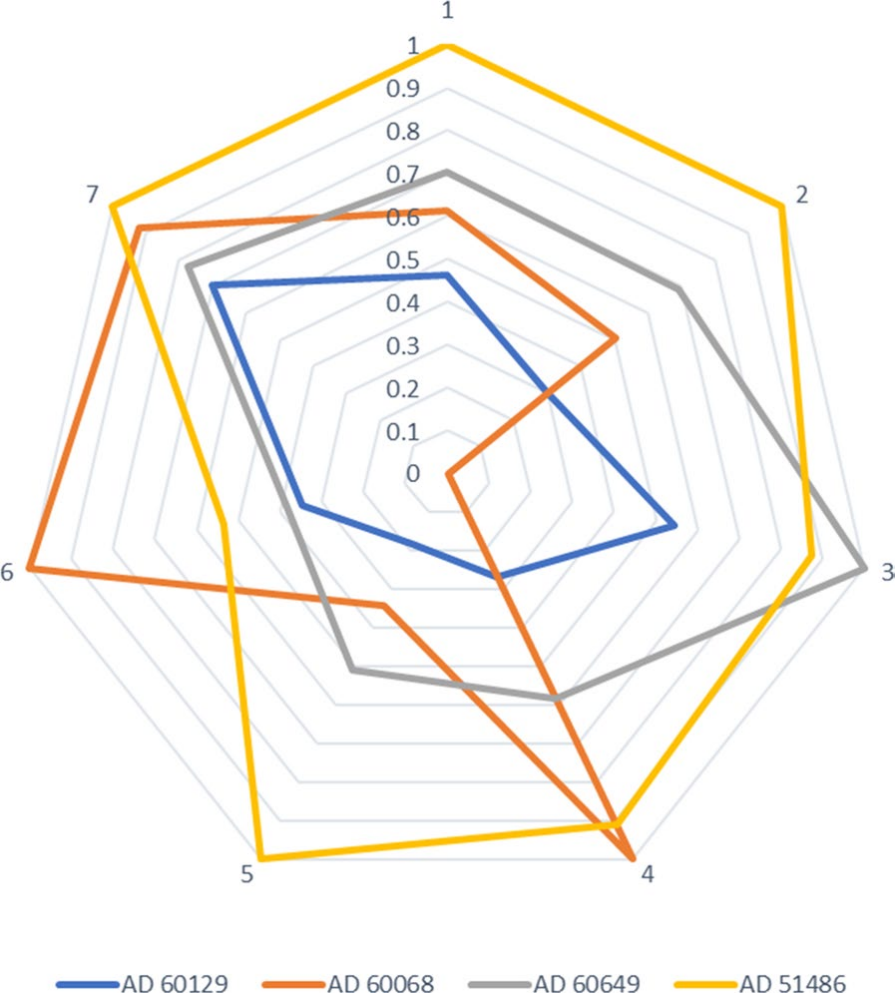
Diagrams depicting pathological features generated by each AD inocula. Each parameter measured in AD brain treated mice were normalized to the higher value among all inocula. These parameters include 4G8 staining in cortex (1), 4G8 staining in hippocampus (2), vascular amyloid deposition (3), ThS staining in cortex (4), ThS staining in hippocampus (5), ThS/4G8 staining in cortex (6) and ThS/4G8 staining in hippocampus (7)

As mentioned, prion strains accumulate in the brain in different patterns, displaying variable anatomical tropisms and deposits, including diffuse, compact, extra- and intra-cellular inclusions [53]. Similar variation in Aβ deposition has been described in AD [14, 15]. Here, we characterized amyloid deposition in the brain of four individuals clinically diagnosed with AD dementia. These patients showed strikingly different patterns of amyloid deposition in terms of total Aβ levels, cellular location, and type of aggregates (Fig. 1 and Table 2). To partially assess the pathological significance of this assortment of aggregates, we inoculated brain extracts from these patients in susceptible mice [46, 49]. Interestingly, the extent of amyloid induction was not directly associated with the levels of Aβ administered in each case (Fig. 2). In fact, the brain displaying the lowest net amount of Aβ deposits (AD60068) was the one with the highest seeding activity (Fig. 2o). Patient AD60068 displayed small intracellular inclusions that were less compact compared to the ones observed in the other AD brains included in this study. Whether this sample is enriched in oligomeric Aβ species, generally considered as the best seeds [59], will be reported in future studies. In addition, we observed that Aβ pathology in the brains of experimental mice displayed different arrangements as judged by their differential reactivity to ThS (Fig. 4) and tropism to blood vessels (Fig. 3). This additional data set suggests that the pathological information encoded in Aβ seeds is able to propagate different pathological traits. The pathological features generated in treated mice are summarized in Fig. 5. There, it can be clearly appreciated that the four different AD inocula promote the appearance of different patterns of brain amyloidosis, probably attributed to the different arrangements of amyloid deposition present on them. Nevertheless, at this point we cannot rule out the possibility that additional differences in the composition of the AD brains used in this experiment (e.g., differential inflammatory response, variable Aβ_40_/Aβ_42_ ratios as suggested in Fig. 1, composition and distribution of tau pathology, etc.) are responsible for the differences in in vivo seeding. The role of these potential variables in the propagation of Aβ misfolding should be carefully addressed in future studies.

As expected, mice inoculated with the brain extract from an aged non-demented individual displayed the same amount of amyloid deposits compared to non-injected controls (Fig. 2). Nevertheless, a small but significant induction of CAA was observed in mice treated with this particular injectate (Fig. 3). As extensively reported, amyloid deposition is a common event occurring during aging and, in many cases, extensive cerebral amyloid deposition is observed in the absence of clinical signs [60]. As shown in Fig. 1, this “control” brain contains measurable levels of PBS-insoluble Aβ that were actually comparable to the ones measured in one AD-derived sample (AD60068). Importantly, these Aβ aggregates were unable to induce substantial pathology, in line with the absence of clinical symptoms observed in this individual.

In the prion field, prion strain selection and cloning require serial infectivity passages within the same animal species [30, 61, 62]. Assuming that misfolded Aβ is also capable to generate conformational strains, similar approaches could be applied to this AD-linked protein. Nevertheless, pathological induction (seeding efficiencies, vascular tropism, ThS reactivity of the aggregates) in this experiment did not resemble the one observed in the parental brains. This could be explained by the aggressive pathology displayed by APP/PS1 due to the introduction of double AD-linked mutations and APP overexpression [44]. In that sense, we believe that the pathology generated in experimental mice is a combined result of seed-templated deposition and the endogenous pathology generated in them. In that sense, the serial in vivo passage approach used to isolate strains of infectious prions may not apply to APP/PS1 mice. In addition, it is predicted that each AD brain contains not just one, but a combination of different Aβ strains similarly as it has been hypothesized for their prion counterparts [63]. Future research, using less aggressive models of Aβ deposition and isolated Aβ strains should provide a clearer picture on the role of Aβ conformational strain variation in AD.

One limitation of this study is that estimations of seeding activity were not directly performed by injecting the same amount of Aβ peptides but the same mass of brain tissue. Comparisons of seeding efficiency for each inoculum were done indirectly by calculating the ratio between amyloid induction and the estimated amount of Aβ present in each inoculum. Unfortunately, proper comparisons would need to “titrate” the seeding activity of each material by performing seeding experiments with serial dilutions of each injectate as previously described [24]. This will allow us to assess the effect of Aβ and other potential factors present in the brain that could alter the prion-like propagation of these aggregates (e.g., molecules stabilizing Aβ aggregates, inflammatory components making mice’s brains more susceptible for seeding, etc.). In addition, considering that possible misfolded Aβ strains present in each AD brain are not a single but a group of different co-existing conformations, diluting each brain extract to normalize Aβ concentrations may not be accurate. Future experiments exploring the composition, stability and biological function of individual and grouped Aβ strains co-existing in AD brains could provide us with a better understanding of the biological relevance of the Aβ conformational strain phenomenon in AD.

## Conclusions

Our results support the hypothesis that AD includes a spectrum of pathological conditions characterized by the accumulation of misfolded proteins leading to dementia. The identification of Aβ strains could lead to personalized and more effective treatments that may improve AD prognosis.

## Supplementary Information

**Additional file 1**.

**Additional file 2**.

## Data Availability

The datasets used and/or analyzed during the current study are available from the corresponding author on reasonable request.
