# Supplementary material for: Amyloid pathology arrangements in Alzheimer’s disease brains modulate in vivo seeding capability
Source: Acta Neuropathol Commun. 2021 Mar 30;9:56. doi: 10.1186/s40478-021-01155-0 (PMC8008576; doi:10.1186/s40478-021-01155-0)

Supplemental Table 1. Incidence of vascular amyloidosis in the hippocampus of APP/PS1 mice treated with AD and control brain extracts.

Inoculum	Incidence of vascular amyloidosis (% affected mice)
60129	0%
60068	0%
60649	17%
51486	33%
58652	0%

Supplemental Figure 1

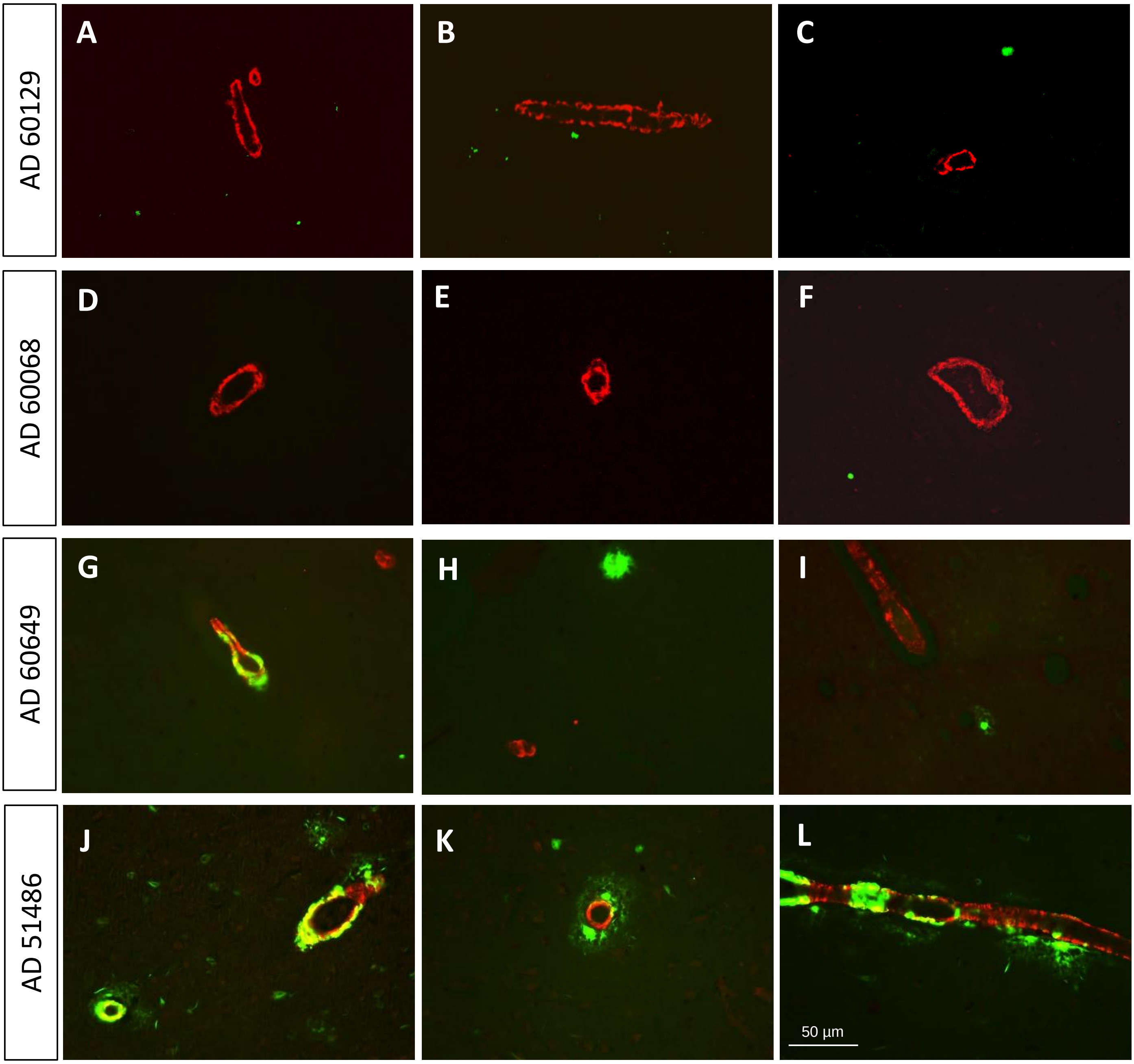

Supplement: Supplementary file 1 — Additional file 1. [file 40478_2021_1155_MOESM1_ESM.pdf]
