# Supplementary material for: Amyloid pathology arrangements in Alzheimer’s disease brains modulate in vivo seeding capability
Source: Acta Neuropathol Commun. 2021 Mar 30;9:56. doi: 10.1186/s40478-021-01155-0 (PMC8008576; doi:10.1186/s40478-021-01155-0)
Supplement: Supplementary file 2 — Additional file 2. [file 40478_2021_1155_MOESM2_ESM.pdf]

SUPPLEMENTAL FIGURE LEGENDS

Supplemental Figure 1. Vascular Aβ deposition in brain tissue from AD patients. Double staining with ThS (green) and anti-SMA antibody (red) confirm the absence of vascular Aβ deposits in brain samples from patients AD60129 (A-C) and AD60068 (G-I). Vascular Aβ was rare in sample AD60649 (G-I) (a single event in all brain slices analyzed) and abundant in patient AD51486 (J-L). Scale bar in L depicts 50 μm and applies to all panels.
